# Small-Sized Mg–Al LDH Nanosheets Supported on Silica Aerogel with Large Pore Channels: Textural Properties and Basic Catalytic Performance after Activation

**DOI:** 10.3390/nano8020113

**Published:** 2018-02-16

**Authors:** Lijun Wang, Yusen Wang, Xiaoxia Wang, Xiaolan Feng, Xiao Ye, Jie Fu

**Affiliations:** School of Chemistry and Chemical Engineering, Shaoxing University, Shaoxing 312000, China; wys0_0@163.com (Y.W.); xxwang0119@163.com (X.W.); xlfeng672@163.com (X.F.); lambor12146233yx@163.com (X.Y.); starry1874fj@163.com (J.F.)

**Keywords:** layered double hydroxides, nanocatalysts, mesoporous silica, support, solid base

## Abstract

Layered double hydroxides (LDHs) have been widely used as an important subset of solid base catalysts. However, developing low-cost, small-sized LDH nanoparticles with enhanced surface catalytic sites remains a challenge. In this work, silica aerogel (SA)-supported, small-sized Mg–Al LDH nanosheets were successfully prepared by one-pot coprecipitation of Mg and Al ions in an alkaline suspension of crushed silica aerogel. The supported LDH nanosheets were uniformly dispersed in the SA substrate with the smallest average radial diameter of 19.2 nm and the thinnest average thickness of 3.2 nm, both dimensions being significantly less than those of the vast majority of LDH nanoparticles reported. The SA/LDH composites also showed large pore volume (up to 1.3 cm^3^·g) and pore diameter (>9 nm), and therefore allow efficient access of reactants to the edge catalytic sites of LDH nanosheets. In a base-catalyzed Henry reaction of benzaldehyde with nitromethane, the SA/LDH catalysts showed high reactant conversions and favorable stability in 6 successive cycles of reactions. The low cost of the SA carrier and LDH precursors, easy preparation method, and excellent catalytic properties make these SA/LDH composites a competitive example of solid-base catalysts.

## 1. Introduction

Layered double hydroxides (LDHs) are a class of two-dimensional anionic clays composed of positively-charged layers of mixed bivalent and trivalent metal hydroxides with charge-balancing anions and hydrogen-bonded water molecules in the interlayer [1,2,3,4]. Mg–Al LDHs, represented by the general formula [Mg^2+^_1–*x*_Al^3+^*_x_*(OH)_2_]*^x^*^+^(A*^n^*^–^)*_x_*_/*n*_·*m*H_2_O, have been widely used as an important subset of solid base catalysts [5,6,7,8,9,10]. In particular, calcined Mg–Al LDHs composed of uniformly-dispersed magnesium–aluminum mixed oxides with variable proportions have shown very attractive properties as catalysts for a broad spectrum of base-catalyzed organic reactions, including transesterification [11,12], isomerization of glucose [13,14,15], Henry reactions [16], Cannizzaro reactions [17], epoxidation of olefins [18], alkylation of phenol [19], and aldol condensation [20,21,22].

For flake-like LDH, the activated catalytic sites are mainly located at their edges [23,24,25,26]. Generally, LDH flakes with a smaller size (especially the radial size) possess higher surface area, corresponding with more edge catalytic sites [27,28]. However, these smaller LDH nanoparticles are readily agglomerated by *ab*-face stacking in solution due to their high charge density and hydrophilicity [29,30,31]. Furthermore, small LDH nanoparticles are hard to separate after preparation and reaction in liquid systems [28]. A good way to solve these problems is to support the LDH nanoparticles on high-surface-area carriers. A variety of materials such as SBA-15 [28,32], carbon nanofibers [27], multi-walled carbon nanotubes [33], graphene oxide [34,35], mesoporous AlOOH [36], zeolite [37], hollow carbon nanospheres [38,39], silica nanofiber [1], γ-Fe_2_O_3_ [40], SiO_2_ nanospheres [30,41,42,43,44,45,46], biochar [47], Fe_3_O_4_ [48], cellulose [49], Cu nanowires [50], and magnesium ferrite nanoparticles [51] have been employed as carriers to prepare supported LDHs. The core/shell structured silica-sphere-supported LDHs have been especially studied by several groups [30,41,42,43,44,45,46] very recently due to their easy preparation and excellent properties such as tunable size, crystallinity, and morphology of LDH. However, there is still a lack of extensive research of mesoporous silica (mSiO_2_)-supported LDH systems where mSiO_2_ can provide more surface area, far more than that of silica nanosphere, for the depositing of LDH nanoparticles. This may be attributed to two reasons: (1) In the mSiO_2_-supported HT structure, the supported LDH nanoparticles located at the inner surface of mSiO_2_ would block the pore channels, limiting the accessibility of active sites of the confined LDH; (2) The higher cost and more complex preparation of mSiO_2_ compared with the SiO_2_ sphere usually synthesized by a simple Stöber method. Therefore, developing novel, low-cost, facile mSiO_2_-supported LDHs with small size and easily accessible edge sites are expected.

Silica aerogels (SAs) have attracted much attention in science and technology due to their high surface area and large pore volume [52,53]. Moreover, SAs are important low-cost modern industrial materials for heat-insulation and heat-resistance applications. In this work, low-cost commercial SAs with a large average pore diameter of 34.4 nm and large pore volume of 5.92 cm^3^·g were selected as carriers to prepare supported Mg–Al LDH nanoparticles by a one-pot coprecipitation method. The large pore diameter and pore volume facilitate the nucleation and growth of LDH nanoparticles at the inner surface of SA and the minimization of pore blocking by the inner LDH particles. As expected, the obtained SA/LDH composites showed large average pore diameters (>9 nm), large pore volume (up to 1.3 cm^3^·g), and high surface area (up to 587.4 m^2^·g) and could therefore provide easy access of reactants to the edge catalytic sites of the incorporated LDH nanosheets. In this context, the morphology, textural properties, and base site strength of a series of SA/LDH composites synthesized at different Mg/Al ratios and hydrothermal temperatures were characterized by TEM, XRD, EDS, BET, and CO_2_-TPD. A base-catalytic Henry reaction of benzaldehyde with nitromethane was employed as a model reaction to evaluate the catalytic properties of the supported LDHs using the unsupported LDH nanosheets and bare SA as control samples.

## 2. Materials and Methods 

### 2.1. Materials

Magnesium nitrate, aluminium nitrate, sodium hydrogen carbonate, nitromethane, ethanol, dichloromethane, toluene, *N*,*N*-Dimethylformamide, tetrahydrofuran, and sodium hydroxide were all of analytical grade and purchased from Aladdin Reagent Co., Ltd. (Shanghai, China). SA was provided by Nano Tech Co., Ltd. (Shaoxing, China). Benzaldehyde (>98.0%, GC) and o-xylene (>98.0%, GC) were purchased from TCI (Tokyo, Japan).

### 2.2. Experimental

The SA powder was calcined at 823 K for 2 h to remove surface organic groups. The treated SA powder (500 mg) was suspended in 500 mL of deionized water by sonication for 30 min. 10 mL of mixed solution A (*m* mol·L^−1^ Mg(NO_3_)_2_·6H_2_O and *n* mol·L^−1^ Al(NO_3_)_3_·9H_2_O) was added to the SA suspension and the obtained mixture stirred for 30min. The pH of the solution was adjusted to 8.8 with the mixed solution B (NaOH/NaHCO_3_ (0.5 M/0.5 M)). 140 mL of mixed solution A and various amounts of solution B were added alternately to maintain the pH at 8.8, followed by hydrothermal treatment at 80 °C, 105 °C, or 150 °C for 24 h. The Mg:Al ratio was varied such that *m* + *n* = 0.05 mol·L^−1^ and *m:n* = 3:1, 5:2, and 2:1. The obtained products were concentrated by centrifugation, decanted and washed three times with deionized water, and vacuum-dried. The final samples were denoted as SA/LDH-Mg*_x_*Al-*y*, where *x* and *y* represent the Mg/Al mole ratio in the precursor solution and the hydrothermal temperature, respectively. For the control sample of unsupported LDH nanosheets (LDH-Mg_2_Al-80), the synthetic method was the same as for the SA/LDH composite except for using a pH of 9.5 instead of 8.8 so as to obtain LDH with an Mg/Al ratio of ~2:1. All materials were calcined at 723 K for 2 h and cooled under a flowing stream of nitrogen before catalyst testing.

### 2.3. Characterization

Transmission electron micrographs were taken using a JEOL JEM-2100 Transmission Electron Microscope (TEM) (JEOL Ltd., Tokyo, Japan) operating at an accelerating voltage of 200 kV. Scanning electron micrographs and energy dispersive spectra were obtained using a JEOL JSM-6360LV scanning electron microscope (SEM) equipped with an X-act energy-dispersive X-ray (EDX) analyzer (Oxford INCA, Oxford Instruments, Abingdon, Oxfordshire, UK). N_2_ adsorption–desorption isotherms were obtained using a Micromeritics ASAP TriStar II 3020 pore analyzer (Micromeritics Instrument Corp., Norcross, GA, USA) at 77 K under continuous adsorption conditions. The samples were outgassed at 150 °C for 8 h before measurements. The specific surface areas were calculated by the Brunauer–Emmett–Teller (BET) method, and pore size distributions were measured using Barrett–Joyner–Halenda (BJH) analysis from the desorption branches of the nitrogen isotherms. X-ray diffraction (XRD) (Rigaku Corp., Tokyo, Japan) patterns were collected using a Rigaku D/Max-2200 PC X-ray diffractometer with a Cu target (40 kV, 40 mA). CO_2_ and NH_3_ temperature-programmed desorption (CO_2_-TPD and NH_3_-TPD) profiles of the (supported) LDH samples were obtained using a Quantachrome ChemBET Pulsar equipped with a thermal conductivity detector (TCD) (Quantachrome Instruments, Boynton Beach, FL, USA). A 100 mg sample was calcined for 2 h at 773 K under flowing helium, and then cooled to 323 K to adsorb NH_3_ or CO_2_. The physical adsorbed CO_2_ was purged by flowing helium at 323 K, and NH_3_- and CO_2_-TPD was subsequently carried out at the rate of 10 K min^−1^ up to 1073 K. The Mg/Al and Mg/Al/Si mole ratios of the prepared series of SA/LDH composites were assessed by semiquantitative EDS analysis. Mg contents in fresh and used SA/LDH-Mg_2_Al-80 and the Mg/Al mole ratio of unsupported LDH were determined using inductively coupled plasma atomic emission spectroscopy (Leeman Prodigy XP ICP-AES spectrometer) (Teledyne Leeman Labs, Hudson, NH, USA).

### 2.4. Catalytic Evaluation

The Henry reaction of benzaldehyde with nitromethane was performed in liquid phase under atmospheric pressure to evaluate the catalytic properties of the SA/LDH composite series. Benzaldehyde (1 mmol), o-xylene (0.1 g, internal standard), nitromethane (5 mL), catalyst (50 mg), and a magnetic stir bar were placed into a 50 mL Schott bottle. The bottle was then sealed and put into a magnetic stirring bath (equipped with digital heating). The reaction started when the temperature reached 80 °C. After stirring at 400 rpm for 6 h, the reaction was stopped by cooling the bottle to room temperature. The solid catalyst was separated from the reaction product by centrifugation. The reaction product was analyzed by a Shimadzu GC2010 gas chromatograph (GC) (Shimadzu Corp., Kyoto, Japan) equipped with a flame ignition detector (FID), an AOC-20I autosampler, and a capillary column (Rtx-5, 30 m × 0.25 mm × 0.25 μm, crossbond 5% diphenyl/95% dimethyl polysiloxane). For 6 successive cycles of reactions, the used catalyst was washed with ethanol or water to partly remove the carbonaceous products adsorbed and then vacuum-dried for reuse in the first five cycles and calcined at 823 K to thoroughly remove the carbonaceous products for reuse in the sixth (last) cycle. To evaluate the effect of solvents on the catalytic properties of the SA/LDH catalyst, benzaldehyde (1 mmol), o-xylene (0.1 g, internal standard), nitromethane (5 mmol), solvent (4.73 mL), and catalyst (50 mg) were adopted.

## 3. Results and Discussion

**Scheme 1:**
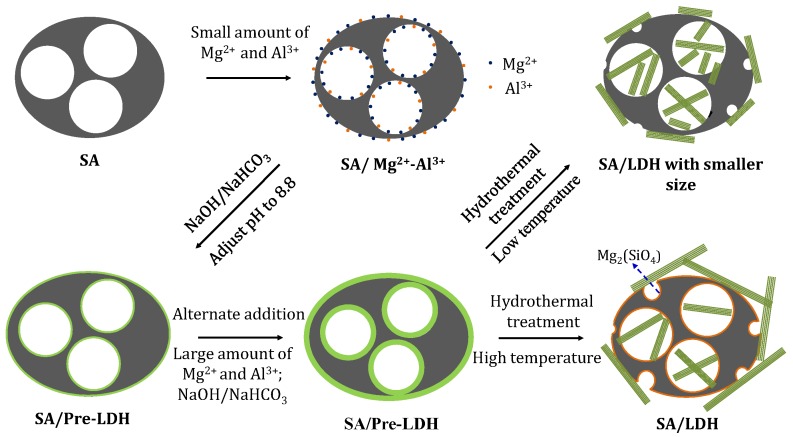
A schematic illustration of the fabrication of silica aerogel (SA)- supported layered double hydroxide (LDH) nanosheets.

The synthesis of SA-supported LDH nanosheets is described in Scheme 1. Briefly, a small amount of mixed solution A (Mg(NO_3_)_2_/Al(NO_3_)_3_) was first added to the SA suspension, in which process the Mg^2+^ and Al^3+^ were adsorbed onto the negatively charged inner and outer surfaces of the SA. Then solution B (NaOH/NaHCO_3_) was added to precipitate the Mg^2+^ and Al^3+^ adsorbed in situ and form LDH precursor (Pre-LDH) uniformly on the surface of the SA. Thereafter, a large amount of mixed solution A and a calculated amount of solution B were added alternately to maintain the solution pH at a certain value. Finally, the SA/LDH composite formed through hydrothermal treatment of the obtained SA-supported Pre-LDH.

**Figure 1:**
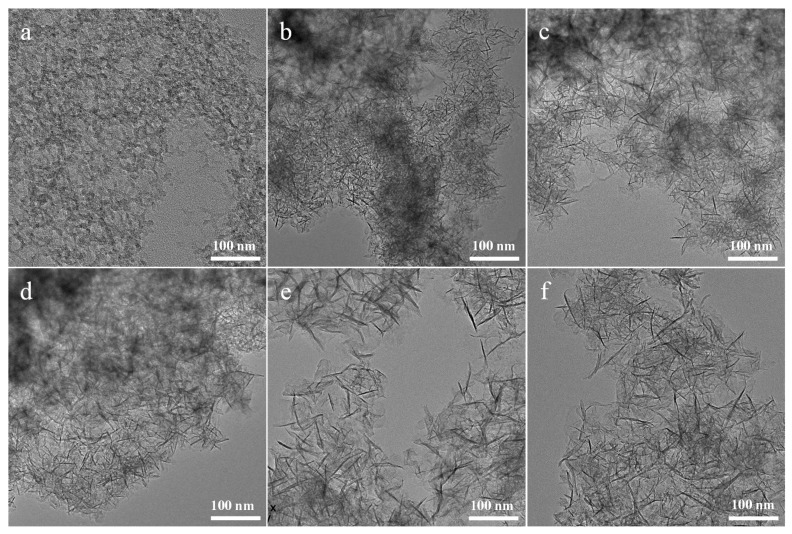
TEM images of SA (**a**); SA/LDH-Mg_2_Al-80 (**b**); SA/LDH-Mg_5_Al_2_-80 (**c**); SA/LDH-Mg_3_Al-80 (**d**); SA/LDH-Mg_2_Al-105 (**e**); and SA/LDH-Mg_2_Al-150 (**f**).

The morphology of initial template SA and the prepared SA/LDH composite were characterized by TEM. Typically, SA shows disordered pore structure (Figure 1a) and low TEM contrast due to its low density and amorphous structure. In the current SA/LDH composite series (Figure 1b–f), a large amount of LDH nanosheets was observed located at the inner and outer surfaces of the SA substrate. The SA/LDH series synthesized at a low temperature of 80 °C all showed small LDH radial diameters and the average radial diameters were *ca* 19.2 nm, 31.4 nm, and 35.8 nm for SA/LDH-Mg_2_Al-80, SA/LDH-Mg_5_Al_2_-80, and SA/LDH-Mg_3_Al-80, respectively (Table 1 and Figure S1). However, the radial diameters of LDH increased to ~53.0 and 57.4 nm for the SA/LDH-Mg_2_Al-105 and SA/LDH-Mg_2_Al-150, which were prepared at higher temperatures of 105 °C and 150 °C, respectively. The same trend was found in the thickness change of the SA/LDH series (Table 1 and Figure S2). The thicknesses were 3.2 nm, 3.2 nm, and 3.6 nm for SA/LDH-Mg_2_Al-80, SA/LDH-Mg_5_Al_2_-80, and SA/LDH-Mg_3_Al-80, respectively, and increased to 4.5 and 4.3 nm, for SA/LDH-Mg_2_Al-105 and SA/LDH-Mg_2_Al-150, respectively. It should be noted that the SA/LDH composite synthesized at higher pH values such as 9.5 showed significant destruction of SA structure due to the strong alkali corrosion in the preparation. Therefore, the impact of pH values on the structure of the composite is not discussed in this work. EDS mappings of the prepared SA/LDH composites (Figure S3) showed an almost complete overlap of the Si, Mg, Al, O elemental mappings, further demonstrating that the supported LDH nanosheets were uniformly dispersed on the SA substrate. However, the Mg/Al ratios in the obtained composites were not consistent with those in precursor solutions (Table 1) as only a fraction of the Mg(II) ions precipitated at the synthetic pH of 8.8 due to the high solubility product constant (*K_sp_* = 5.1 × 10^−12^) of Mg(OH)_2_ while Al(III) almost completely precipitated in the form of Al(OH)_3_ with a low *K_sp_* value of 1.3 × 10^−33^ under the same conditions. In addition, the Mg/Al mole ratios of SA/LDH-Mg_2_Al-105 and SA/LDH-Mg_2_Al-150 were both higher than that of SA/LDH-Mg_2_Al-80 which is attributed to the formation of magnesium silicate under the high synthetic temperatures as proven by the subsequent XRD analysis.

**Figure 2:**
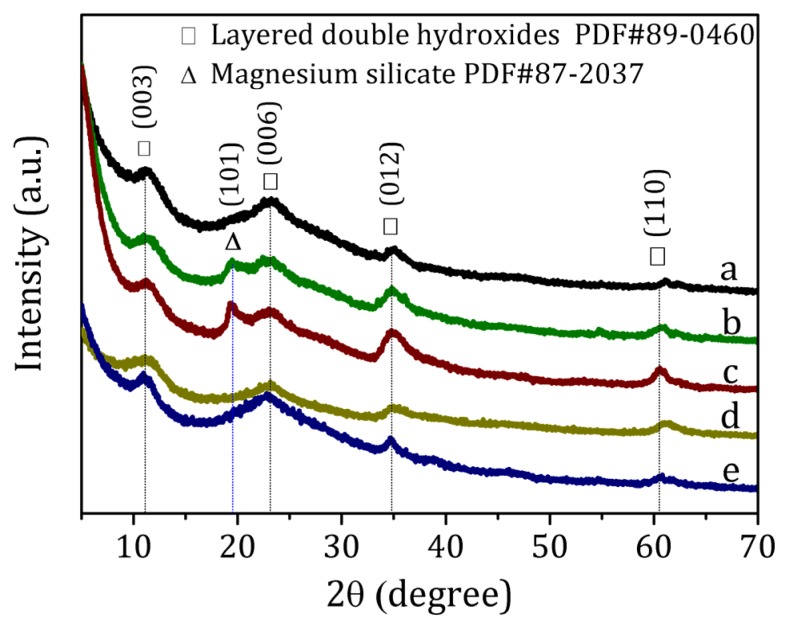
XRD patterns of SA/LDH-Mg_2_Al-80 (**a**); SA/LDH-Mg_2_Al-105 (**b**); SA/LDH-Mg_2_Al-150 (**c**); SA/LDH-Mg_5_Al_2_-80 (**d**); and SA/LDH-Mg_3_Al-80 (**e**).

The XRD pattern of the fabricated SA/LDH composites (Figure 2) all showed typical but weak LDH diffraction peaks (Standard PDF card: #89-0460), which can be indexed to the (003), (006), (012), and (110) planes. For the SA/LDH prepared at a high synthetic temperature of 105 °C and 150 °C, the strongest reflection peak of magnesium silicate indexed to the (101) plane (Standard PDF card: #87-2037) appeared, which is probably because SiO_3_^2−^ derived from the partial dissolution of SA in an alkaline preparation solution of SA/LDH tends to react with unprecipitated Mg(II) ions in the LDH precursor at high synthetic temperatures.

**Figure 3:**
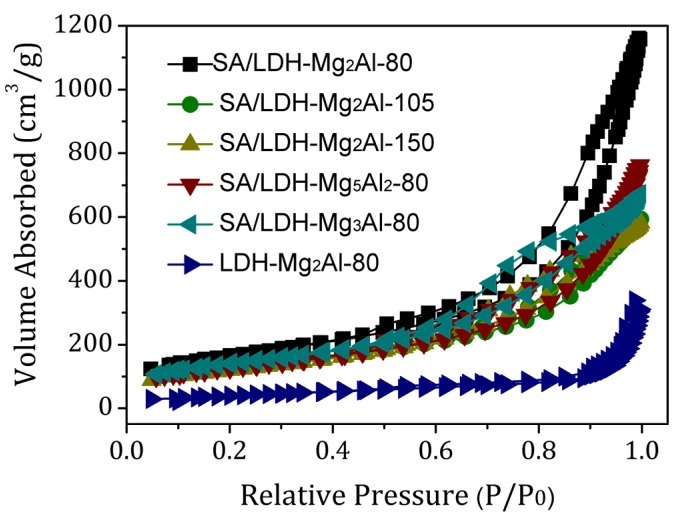
N_2_ adsorption–desorption isotherms of SA/LDH-Mg_2_Al-80, SA/LDH-Mg_2_Al-105, SA/LDH-Mg_2_Al-150, SA/LDH-Mg_5_Al_2_-80, and SA/LDH-Mg_3_Al-80.

Figure 3 and Figure S4 show the N_2_ adsorption–desorption isotherms of the SA/LDH composites, SA and the unsupported LDH, respectively. All SA/LDH composites displayed a type IV isotherm with H_1_ hysteresis and a sharp increase in volume adsorbed at P/P_0_ ≈ 0.6 to 0.7, which indicates that SA/LDH composite partially retained the mesoporous structure of the SA. The BET surface area (S_BET_), pore volume (V_p_), and pore diameter (D_p_) of the relative samples determined via nitrogen adsorption–desorption measurements are listed in Table 1. All SA/LDH samples showed a high surface area of more than 400 m^2^·g, a large pore volume of greater than 0.90 cm^3^·g, and pore diameters larger than 9 nm. SA/LDH-Mg_2_Al-80 possesses an obviously higher surface area than the other SA/LDH samples, and the surface area of the samples decreased with increasing synthetic temperature. As expected, the surface area, pore volume, and pore diameter of the SA/LDH series all decreased in comparison to the substrate SA due to the partial blockage of SA pore channels by LDH nanosheets and the inherently higher density of the LDH than that of the porous SA in the composite. On the basis of the results in Table 1, it is concluded that the SA/LDH composites with smaller LDH sizes have the higher surface area, which is attributed to the smaller LDH nanosheets minimizing the blocking of SA pore channels.

**Figure 4:**
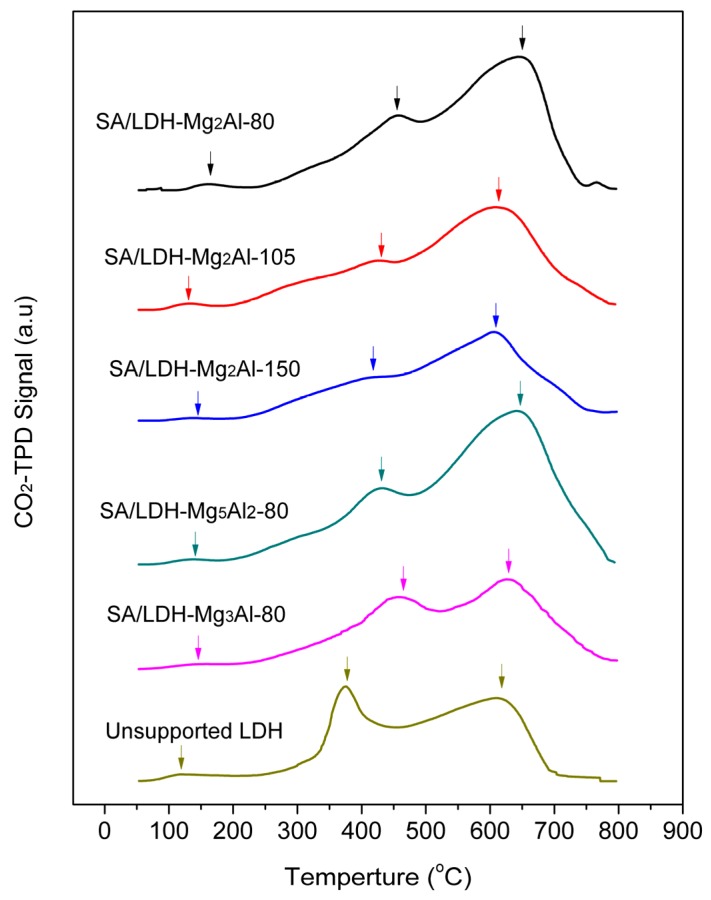
CO_2_-TPD profiles of SA/LDH series and the unsupported LDH nanosheets.

To evaluate the basicity of the SA/LDH series, CO_2_-TPD measurements were conducted. In general, activated LDHs possess weakly basic sites, moderately basic sites, and strongly basic sites, ascribed to the surface hydroxyl group, metal–oxygen pairs (e.g., Mg^2+^–O_2_), and low-coordination oxygen anions, respectively [54]. For CO_2_-TPD, the basic sites in the high desorption temperature range are usually designated as strong basic sites, and vice versa [55]. As shown in Figure 4 and Table 2, the SA/LDH series showed a broad CO_2_ desorption band from 230 to 790 °C with the highest peak in the high temperature range of 605.7 to 649.3 °C and a broad shoulder peak in the low temperature range of 417.6 to 458.5 °C, demonstrating that the surface basic strength of the composites was widely distributed and the SA/LDH composites possessed mainly moderate and strong base sites originating from the O^2−^ anions and surface Mg–O^2−^ pairs of LDH. The highest desorption peak position of SA/LDH-Mg_2_Al-80, SA/LDH-Mg_5_Al_2_-80, and SA/LDH-Mg_3_Al-80 all shifted to higher temperatures compared with SA/LDH-Mg_2_Al-105, SA/LDH-Mg_2_Al-150, and LDH-Mg_2_Al-80, indicating that the SA/LDH samples synthesized at low temperature have more strongly basic sites than those synthesized at high temperatures and the unsupported LDH with large size and some particle aggregation (Figure S5). In addition, all samples showed a relatively weak peak in the temperature range 121.5 to 160.6 °C, corresponding to weakly basic sites derived from the OH^-^ groups of the LDH. The total peak area of CO_2_-TPD signals is linearly proportional to the amount of CO_2_ adsorbed and can thus be used as a semi-quantitative parameter for assessing the number of basic sites of the catalysts. The basic site numbers of the samples are ranked as follows (Table 2): SA/LDH-Mg_5_Al_2_-80 > SA/LDH-Mg_2_Al-80 > SA/LDH-Mg_3_Al-80 > LDH-Mg_2_Al-80 > SA/LDH-Mg_2_Al-105 > SA/LDH-Mg_2_Al-150. The higher basic site number of SA/LDH-Mg_5_Al_2_-80 in comparison to that of SA/LDH-Mg_2_Al-80, which has a smaller LDH size and a higher surface area, may be due to the higher Mg content of the former than the latter. This discrepancy could also explain the higher observed basic site density of the unsupported LDH in comparison to those of the SA/LDH-Mg_2_Al-105 and SA/LDH-Mg_2_Al-150 with greater surface area and smaller LDH size. From these results, it is concluded that the basicity of LDH in this system is enhanced by multiple factors including small LDH size, high surface area, and high Mg content.

The base-catalyzed Henry reaction of conversion of benzaldehyde to *trans*-1-phenyl-2-nitro-ethanol and *trans*-1-nitro-2-phenylethylene (Species 1, 2, and 3 in Equation (1), respectively) was taken as a model reaction to evaluate the basic catalytic properties of the SA/LDH composites. The solvent plays a significant role in catalysis reactions, such as dissolution of reactants and products, adjusting the interaction of reactants and products with the surface catalytic sites of catalysts and activating reactants and intermediates. The catalytic properties of SA/LDH-Mg_2_Al-80 with the smallest LDH size in different solvents were given in Table 3. In general, the relatively high conversions were obtained in the solvents with relative weak polarity, such as nitromethane, dichloromethane, and toluene, while the solvents with strong polarity, such as ethanol, THF, DMF, and water, gave low conversions. This trend was similar to that found in the reaction of nitrobenzaldehyde with nitromethane [56] and could be attributed as the reason that protic polar solvents competed with the nitromethane (pKa = 10.21) for the surface basic sites of the catalyst, reducing the number of catalytic sites available for the reaction. When nitromethane was used as both a solvent and a reactant, the highest conversion of 96.8% was obtained owing to the higher concentration of nitromethane and thus a higher contact probability of nitromethane with benzaldehyde. In addition, the adsorption of the yellow product *trans*-1-nitro-2-phenylethylene on the catalyst was found for the reactions in all solvents except for ethanol (Figure S6). Based on these results and the low environment hazard, nitromethane was selected as the solvent for the subsequent studies.
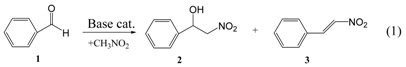


The catalytic properties of SA/LDH series catalysts with various textures are given in Table 4. SA/LDH-Mg_2_Al-80, SA/LDH-Mg_5_Al_2_-80, and SA/LDH-Mg_3_Al-80 synthesized at a low temperature all showed high reaction conversions of 96.8%, 98.2%, and 92.7%, respectively, which are significantly higher than those of SA/LDH-Mg_2_Al-105 (59.3%) and SA/LDH-Mg_2_Al-150 (51.9%) synthesized at high temperatures (Table 4), demonstrating that the reactant conversions are positively correlated with the basicity of the catalysts. It should be noted that the SA/LDH-Mg_2_Al-80 has a selectivity of 97.3% to product 3, which is significantly higher than those of the SA/LDH-Mg_5_Al_2_-80 (78.6%) and SA/LDH-Mg_3_Al-80 (87.2%), which could be attributed to the higher Al content of SA/LDH-Mg_2_Al-80, endowing it with higher acidic site density and acidic strength than SA/LDH-Mg_5_Al_2_-80 and SA/LDH-Mg_3_Al-80 as shown by NH_3_-TPD (Figure S7 and Table S1). This characteristic would promote the dehydration of *trans*-1-phenyl-2-nitro-ethanol to give *trans*-1-nitro-2-phenylethylene [57]. This reaction did not proceed when using bare SA as a catalyst, as expected, and the unsupported LDH-Mg_2_Al-80 showed a reactant conversion of 75.9%, lower than SA/LDH-Mg_2_Al-80, which is attributed to the higher basic strength of SA/LDH-Mg_2_Al-80 than LDH-Mg_2_Al-80. Summarizing the results above, the SA/LDH samples with high basicity all showed high reactant conversions (>90%) in the model Henry reaction, indicating the favorable accessibility of the surface basic sites of SA/LDH composite.

**Figure 5:**
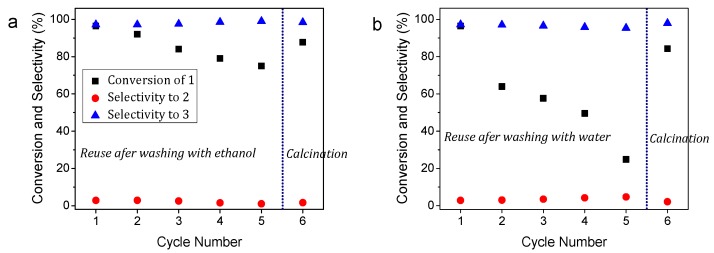
Catalytic capacities of SA/LDH-Mg_2_Al-80 in 6 successive cycles of reactions: (**a**) The used catalyst was refreshed by ethanol washing in the first 5 cycles and calcination in the sixth cycle; (**b**) The used catalyst was refreshed by water washing in the first 5 cycles and calcination in the sixth cycle. Reaction conditions: catalyst, 50 mg; benzaldehyde, 1 mmol; o-xylene, 0.1 g; nitromethane, 5 mL; time, 6 h; reaction temperature, 80 °C.

To examine the stability of the SA/LDH catalyst against deactivation, 6 successive cycles of reactions of benzaldehyde with nitromethane over SA/LDH-Mg_2_Al-80 were conducted using nitromethane as a solvent. After each cycle, the used catalyst was washed with ethanol or water to remove the products adsorbed. For the last cycle, the carbonaceous products on the used catalyst were thoroughly removed by calcination before the reaction. In the first five cycles, the conversions decreased continuously from 96.4% to 75% for the catalyst reused by ethanol washing treatment, while those declined sharply to 24.8% for the catalyst reused by water washing treatment (Figure 5). The contents of the active component Mg are 21.9% and 21.2% in the fresh catalyst and used catalyst after six cycles (ethanol washing), respectively, determined by ICP-MS, demonstrating a negligible leaching of Mg^2+^ ions. Therefore, it could be concluded that catalyst deactivation was mainly derived from the covering of carbonaceous products on the surface active sites of the catalyst and ethanol washing could effectively remove the carbonaceous products adsorbed on the catalyst and prevent the deactivation of catalysts, which is consistent with the above observation of a weak adsorption of the yellow product on the catalyst when using ethanol as a solution. In the sixth cycle, after calcining the used catalysts, the conversions restored from 75% to 87.7% and from 24.8% to 84.3% for the ethanol-washing sample and the water-washing sample, respectively. The high selectivities to product *trans*-1-nitro-2-phenylethylene were kept in all cycles in spite of the obvious changes of the conversions.

Various solid base catalysts have been employed for catalyzing the Henry reaction of benzaldehyde with nitromethane, and different reaction conditions were adopted based on the inherent characteristics of the special catalysts. Table 5 gives a rough catalytic property comparison of the SA/LDH catalysts with other solid base catalysts regardless of the specific reaction conditions. The SA/LDH catalysts showed the highest conversion of 98.2%, which is higher than those of most of the other inorganic catalysts and comparable to those of the catalysts containing organic base groups with high cost and low thermal stability. Furthermore, compared to other inorganic catalysts, the optimized SA/LDH catalyst gave a higher selectivity of 97.3% to the main product *trans*-1-nitro-2-phenylethylene, which is an important chemical intermediate for slimicides and dyes [57].

## 4. Conclusions

In this work, small-sized Mg–Al LDH nanosheets supported on silica aerogel with a large average pore diameter and large pore volume were successfully prepared by a one-pot coprecipitation method. The impact of synthesis parameters on the textural properties of the obtained composite was evaluated. Low Mg/Al precursor ratios and low hydrothermal treatment temperatures result in small LDH sizes and high surface area of the SA /LDH composite. The SA/LDH composite synthesized at a hydrothermal treatment temperature of 80 °C using an Mg/Al precursor ratio of 2:1 showed the highest surface area of 587.4 m^2^·g and the smallest LDH size with an average lateral dimension of *ca* 19.2 nm and an average thickness of *ca* 3.2 nm. These dimensions are significantly lower than those of the vast majority of (supported) LDH nanoparticles reported. The CO_2_-TPD results revealed that the basicity of LDH in this system is enhanced by multiple factors, including small LDH sizes and high Mg contents of the catalysts. However, these factors may conflict. For example, the sample with the highest Mg content did not exhibit the smallest size of LDH and careful consideration of the implications of optimizing one parameter over the other would be needed. In the base-catalyzed model reaction of benzaldehyde with nitromethane, the activated SA/LDH samples with smaller LDH sizes showed higher reactant conversions (>90%) than those with larger LDH sizes and unsupported LDH nanosheets with some particle aggregation. The used SA/LDH catalysts could be refreshed effectively through ethanol washing or calcination to partly or thoroughly remove the carbonaceous products adsorbed. All these results demonstrate high surface basic site strength and favorable accessibility of the surface catalytic sites of the silica-aerogel-supported, ultra-small LDH nanosheets. The low cost of commercial SA, easy preparation, and excellent properties make the silica-aerogel-supported LDH composite a competitive candidate for solid-base catalysts.

## Figures and Tables

**Table 1:** Textural properties of the SA/LDH composite and the control samples.

Samples	S_BET_ (m^2^·g)	V_p_ (cm^3^·g)	D_p_ (nm)	D_LDH_ *^a^* (nm)	TH_LDH_ *^b^* (nm)	Mg/Al Ratio	Mg/Al/Si Ratio
SA/LDH-Mg_2_Al-80	587.4	1.30	11.9	19.2	3.2	1.24	0.58:0.46:1
SA/LDH-Mg_2_Al-105	460.5	0.90	9.3	53.0	4.5	1.46	0.69:0.47:1
SA/LDH-Mg_2_Al-150	429.5	0.96	9.0	57.4	4.3	1.51	0.77:0.51:1
SA/LDH-Mg_5_Al_2_-80	456.0	0.98	9.9	31.4	3.2	2.10	0.83:0.39:1
SA/LDH-Mg_3_Al-80	496.1	1.12	9.2	35.8	3.6	2.27	0.78:0.34:1
SA	803.8	5.92	34.4	—	—	—	—
LDH-Mg_2_Al-80	120.6	0.69	21.2	87.1	<20	1.94	—

*^a^* The radial diameter of LDH; *^b^* The thickness of LDH.

**Table 2:** The semi-quantitative results of CO_2_-TPD measurements.

Samples	CO_2_-TPD Peak Position (°C)			Total Peak Area (a.u.) *^a^*
	I	II	III	
SA/LDH-Mg_2_Al-80	160.6	455.6	649.3	86188.7
SA/LDH-Mg_2_Al-105	131.3	430.6	612.0	68865.8
SA/LDH-Mg_2_Al-150	142.9	417.6	605.7	43227.1
SA/LDH-Mg_5_Al_2_-80	140.2	431.8	643.1	92283.9
SA/LDH-Mg_3_Al-80	143.1	458.5	628.9	74727.8
LDH-Mg_2_Al-80	121.5	376.7	616.5	69046.4

*^a^* Total peak area is linearly proportional to the amount of CO_2_ adsorbed.

**Table 3:** Henry reaction of benzaldehyde with nitromethane over SA/LDH-Mg_2_Al-80 in different solvents *^a^*.

Entry	Solvent	Conversion of 1 (%)	Selectivity to 2 (%)	Selectivity to 3 (%)
1	Nitromethane	96.8	2.7	97.3
2	Ethanol	28.4	11.7	88.3
3	Dichloromethane	49.0	6.0	94.0
4	Toluene	41.6	5.0	95.0
5	DMF	5.9	15.8	84.2
6	Water	16.7	32.3	67.7
7	THF	27.7	21.8	78.2

*^a^* Reaction conditions: SA/LDH-Mg_2_Al-80, 50 mg; benzaldehyde, 1 mmol; o-xylene, 0.1 g; nitromethane, 5 mmol; solvent, 4.73 mL; time, 6 h; reaction temperature, 80 °C; SA/LDH-Mg_2_Al-80 was activated by calcination at 723 K for 2 h before use.

**Table 4:** Henry reaction of benzaldehyde with nitromethane over SA/LDH series catalysts *^a^*.

Entry	Catalysts *^b^*	Conversion of 1 (%)	Selectivity to 2 (%)	Selectivity to 3 (%)
1	SA/LDH-Mg_2_Al-80	96.8	2.7	97.3
2	SA/LDH-Mg_2_Al-105	59.3	30.9	69.1
3	SA/LDH-Mg_2_Al-150	51.9	28.1	71.9
4	SA/LDH-Mg_5_Al_2_-80	98.2	21.4	78.6
5	SA/LDH-Mg_3_Al-80	92.7	12.8	87.2
6	SA	Trace	—	Trace
7	LDH-Mg_2_Al-80	75.9	13.6	86.4
8	None	Trace	—	Trace

*^a^* Reaction conditions: catalyst, 50 mg; benzaldehyde, 1 mmol; o-xylene, 0.1 g; nitromethane, 5 mL; time, 6 h; reaction temperature, 80 °C; *^b^* All the catalysts were activated by calcination at 723 K for 2 h.

**Table 5:** Comparison of catalytic capacities of SA/LDH catalysts with other solid base catalysts in Henry reaction of benzaldehyde with nitromethane.

Entry	Catalysts	Conversion of 1 (%)	Yield of 2 (%) *^a^*	Yield of 3 (%) *^a^*	Temp. (°C)	Time (h)	Ref.
1	SA/LDH-Mg_2_Al-80	96.8	2.6	94.2	80	6	—
2	SA/LDH-Mg_5_Al_2_-80	98.2	21.0	77.2	80	6	—
3	CaO	60.1	—	—	102	12	[58]
4	MgO	3.0	2.1	—	65	6	[59]
5	Al_2_O_3_	—	37 *^b^*	—	—	12	[60]
6	MgO	—	51 *^b^*	—	—	8	[60]
7	Modified Mg-Al LDH	—	95 *^b^*	—	—	0.5	[60]
8	Tb-MOF-NH_2_	87.0	—	—	90	24	[61]
9	TMAOH intercalated layered silicate	55.0	41.8	3.74	R.T.	6	[62]
10	silica-alumina-NH_2_	—	—	99.0	100	6	[63]
11	SiO_2_-NH_2_	—	—	37.0	100	6	[63]
12	Si-Zr-Ti/PAI-HFs-NH_2_	80	10.4	69.6	50	4	[64]
13	Mg-NHC	—	74	—	60	12	[65]
14	Diamino modified- LDH/silicate composite	96	0.48	95.52	70	2	[66]

*^a^* Yield = Conversion × Selectivity; *^b^* Isolated yield.

## Supplementary Materials

The following are available online at http://www.mdpi.com/2079-4991/8/2/113/s1, Figure S1: TEM images of SA/LDH-Mg_2_Al-80 (a); SA/LDH-Mg_5_Al_2_-80 (b); SA/LDH-Mg_3_Al-80 (c); SA/LDH-Mg_2_Al-105 (d); and SA/LDH-Mg_2_Al-150 (e) with a large length-to-height ratio of 1.5; the insets are the radial diameter distributions of the supported LDH nanosheets acquired from >150 nanoparticles in the corresponding images, Figure S2: Thickness distributions of the supported LDH nanosheets: SA/LDH-Mg_2_Al-80 (a); SA/LDH-Mg_5_Al_2_-80 (b); SA/LDH-Mg_3_Al-80 (c); SA/LDH-Mg_2_Al-105 (d); and SA/LDH-Mg_2_Al-150 (e) acquired from >150 nanoparticles in Figure S1, Figure S3: EDS mappings and corresponding elemental analysis of SA/LDH-Mg_2_Al-80 (a); SA/LDH-Mg_2_Al-105 (b); SA/LDH-Mg_2_Al-150 (c); SA/LDH-Mg_5_Al_2_-80 (d); and SA/LDH-Mg_3_Al-80 (e); Figure S4: (a) N_2_ adsorption−desorption isotherms and the corresponding BJH pore size distribution (inset) of SA; (b) BJH pore size distributions of the SA/LDH series and the unsupported LDH (LDH-Mg_2_Al-80), Figure S5: TEM image of unsupported LDH nanosheets (LDH-Mg_2_Al-80) using as a contrast sample. The inset is the corresponding radial diameter distribution, Figure S6: The used catalyst SA/LDH-Mg_2_Al-80 collected by centrifugation after the reaction of benzaldehyde with nitromethane in different solvents such as nitromethane, ethanol, dichloromethane, toluene, DMF, Water, and THF, Figure S7: NH_3_-TPD profiles of SA/LDH series synthesized at a temperature of 80 °C, Table S1: The semi-quantitative results of NH_3_-TPD measurements.

## References

[1] Lv W., Mei Q., Fu H., Xiao J., Du M., Zheng Q. (2017). A general strategy for the synthesis of layered double hydroxide nanoscrolls on arbitrary substrates: Its formation and multifunction. J. Mater. Chem. A.

[2] Nishimura S., Takagaki A., Ebitani K. (2013). Characterization, synthesis and catalysis of hydrotalcite-related materials for highly efficient materials transformations. Green Chem..

[3] Li C.M., Wei M., Evans D.G., Duan X. (2014). Layered Double Hydroxide-based Nanomaterials as Highly Efficient Catalysts and Adsorbents. Small.

[4] Wang Q., O’Hare D. (2012). Recent Advances in the Synthesis and Application of Layered Double Hydroxide (LDH) Nanosheets. Chem. Rev..

[5] Sideris P.J., Nielsen U.G., Gan Z.H., Grey C.P. (2008). Mg/Al ordering in layered double hydroxides revealed by multinuclear NMR spectroscopy. Science.

[6] Li L., Ma R.Z., Ebina Y., Iyi N., Sasaki T. (2005). Positively charged nanosheets derived via total delamination of layered double hydroxides. Chem. Mater..

[7] Takehira K., Shishido T., Wang P., Kosaka T., Takaki K. (2004). Autothermal reforming of CH4 over supported Ni catalysts prepared from Mg-Al hydrotalcite-like anionic clay. J. Catal..

[8] Kantam M.L., Choudary B.M., Reddy C.V., Rao K.K., Figueras F. (1998). Aldol and Knoevenagel condensations catalysed by modified Mg-Al hydrotalcite: A solid base as catalyst useful in synthetic organic chemistry. Chem. Commun..

[9] Liu Y., Lotero E., Goodwin J.G., Mo X. (2007). Transesterification of poultry fat with methanol using Mg-A1 hydrotalcite derived catalysts. Appl. Catal. A-Gen..

[10] Fan G.L., Li F., Evans D.G., Duan X. (2014). Catalytic applications of layered double hydroxides: Recent advances and perspectives. Chem. Soc. Rev..

[11] Shumaker J.L., Crofcheck C., Tackett S.A., Santillan-Jimenez E., Morgan T., Ji Y., Crocker M., Toops T.J. (2008). Biodiesel synthesis using calcined layered double hydroxide catalysts. Appl. Catal. B-Environ..

[12] Reyes I.C., Salmones J., Zeifert B., Contreras J.L., Rojas F. (2014). Transesterification of canola oil catalized by calcined Mg-Al hydrotalcite doped with nitratine. Chem. Eng. Sci..

[13] Delidovich I., Palkovits R. (2015). Structure-performance correlations of Mg-Al hydrotalcite catalysts for the isomerization of glucose into fructose. J. Catal..

[14] Yu S., Kim E., Park S., Song I.K., Jung J.C. (2012). Isomerization of glucose into fructose over Mg-Al hydrotalcite catalysts. Catal. Commun..

[15] Lee G., Jeong Y., Takagaki A., Jung J.C. (2014). Sonication assisted rehydration of hydrotalcite catalyst for isomerization of glucose to fructose. J. Mol. Catal. A-Chem..

[16] Bharali D., Devi R., Bharali P., Deka R.C. (2015). Synthesis of high surface area mixed metal oxide from the NiMgAl LDH precursor for nitro-aldol condensation reaction. New J. Chem..

[17] Kikhtyanin O., Lesnik E., Kubicka D. (2016). The occurrence of Cannizzaro reaction over Mg-Al hydrotalcites. Appl. Catal. A-Gen..

[18] Angelescu E., Pavel O.D., Birjega R., Florea M., Zavoianu R. (2008). The impact of the “memory effect” on the catalytic activity of Mg/Al; Mg,Zn/Al; Mg/Al,Ga hydrotalcite-like compounds used as catalysts for cycloxene epoxidation. Appl. Catal. A-Gen..

[19] Wu G.D., Wang X.L., Chen B., Li J.P., Zhao N., Wei W., Sun Y.H. (2007). Fluorine-modified mesoporous Mg-Al mixed oxides: Mild and stable base catalysts for O-methylation of phenol with dimethyl carbonate. Appl. Catal. A-Gen..

[20] Kikhtyanin O., Hora L., Kubicka D. (2015). Unprecedented selectivities in aldol condensation over Mg-Al hydrotalcite in a fixed bed reactor setup. Catal. Commun..

[21] Oka Y., Kuroda Y., Matsuno T., Kamata K., Wada H., Shimojima A., Kuroda K. (2017). Preparation of Mesoporous Basic Oxides through Assembly of Monodispersed Mg-Al Layered Double Hydroxide Nanoparticles. Chem.-Eur. J..

[22] Hora L., Kelbichova V., Kikhtyanin O., Bortnovskiy O., Kubicka D. (2014). Aldol condensation of furfural and acetone over Mg-Al layered double hydroxides and mixed oxides. Catal. Today.

[23] Roelofs J.C.A.A., Lensveld D.J., van Dillen A.J., de Jong K.P. (2001). On the Structure of Activated Hydrotalcites as Solid Base Catalysts for Liquid-Phase Aldol Condensation. J. Catal..

[24] Tichit D., Gérardin C., Durand R., Coq B. (2006). Layered double hydroxides: Precursors for multifunctional catalysts. Top. Catal..

[25] Abelló S., Medina F., Tichit D., Pérez-Ramírez J., Groen J.C., Sueiras J.E., Salagre P., Cesteros Y. (2005). Aldol Condensations Over Reconstructed Mg–Al Hydrotalcites: Structure–Activity Relationships Related to the Rehydration Method. Chem.-Eur. J..

[26] Abello S., Medina F., Tichit D., Perez-Ramirez J., Cesteros Y., Salagre P., Sueiras J.E. (2005). Nanoplatelet-based reconstructed hydrotalcites: Towards more efficient solid base catalysts in aldol condensations. Chem. Commun..

[27] Winter F., van Dillen A.J., de Jong K.P. (2005). Supported hydrotalcites as highly active solid base catalysts. Chem. Commun..

[28] Li L., Shi J. (2008). In situ assembly of layered double hydroxide nano-crystallites within silica mesopores and its high solid base catalytic activity. Chem. Commun..

[29] Chen C., Yang M., Wang Q., Buffet J.-C., O’Hare D. (2014). Synthesis and characterisation of aqueous miscible organic-layered double hydroxides. J. Mater. Chem. A.

[30] Chen C., Felton R., Buffet J.-C., O’Hare D. (2015). Core-shell SiO_2_@LDHs with tuneable size, composition and morphology. Chem. Commun..

[31] Chen C., Wangriya A., Buffet J.-C., O’Hare D. (2015). Tuneable ultra high specific surface area Mg/Al-CO3 layered double hydroxides. Dalton Trans..

[32] Creasey J.J., Parlett C.M.A., Manayil J.C., Isaacs M.A., Wilson K., Lee A.F. (2015). Facile route to conformal hydrotalcite coatings over complex architectures: A hierarchically ordered nanoporous base catalyst for FAME production. Green Chem..

[33] Garcia-Gallastegui A., Iruretagoyena D., Mokhtar M., Asiri A.M., Basahel S.N., Al-Thabaiti S.A., Alyoubi A.O., Chadwick D., Shaffer M.S.P. (2012). Layered double hydroxides supported on multi-walled carbon nanotubes: Preparation and CO_2_ adsorption characteristics. J. Mater. Chem..

[34] Garcia-Gallastegui A., Iruretagoyena D., Gouvea V., Mokhtar M., Asiri A.M., Basahel S.N., Al-Thabaiti S.A., Alyoubi A.O., Chadwick D., Shaffer M.S.P. (2012). Graphene Oxide as Support for Layered Double Hydroxides: Enhancing the CO_2_ Adsorption Capacity. Chem. Mater..

[35] Memon J., Sun J., Meng D., Ouyang W., Memon M.A., Huang Y., Yan S., Geng J. (2014). Synthesis of graphene/Ni-Al layered double hydroxide nanowires and their application as an electrode material for supercapacitors. J. Mater. Chem. A.

[36] Chang Y.-P., Chen Y.-C., Chang P.-H., Chen S.-Y. (2012). Synthesis, Characterization, and CO_2_ Adsorptive Behavior of Mesoporous AlOOH-Supported Layered Hydroxides. ChemSusChem.

[37] Chen C., Byles C.F.H., Buffet J.-C., Rees N.H., Wu Y., O’Hare D. (2016). Core-shell zeolite@aqueous miscible organic-layered double hydroxides. Chem. Sci..

[38] Xu J., Ma C., Cao J., Chen Z. (2017). Facile synthesis of core-shell nanostructured hollow carbon nanospheres@nickel cobalt double hydroxides as high-performance electrode materials for supercapacitors. Dalton Trans..

[39] Xu J., He F., Gai S., Zhang S., Li L., Yang P. (2014). Nitrogen-enriched, double-shelled carbon/layered double hydroxide hollow microspheres for excellent electrochemical performance. Nanoscale.

[40] Yin S., Li J., Zhang H. (2016). Hierarchical hollow nanostructured core@shell recyclable catalysts [gamma]-Fe2O3@LDH@Au25-x for highly efficient alcohol oxidation. Green Chem..

[41] Shirotori M., Nishimura S., Ebitani K. (2017). Fine-crystallized LDHs prepared with SiO_2_ spheres as highly active solid base catalysts. J. Mater. Chem. A.

[42] Kwok W.L.J., Crivoi D.-G., Chen C., Buffet J.-C., O’Hare D. (2018). Silica@layered double hydroxide core-shell hybrid materials. Dalton Trans..

[43] Chen C., Wang P., Lim T.-T., Liu L., Liu S., Xu R. (2013). A facile synthesis of monodispersed hierarchical layered double hydroxide on silica spheres for efficient removal of pharmaceuticals from water. J. Mater. Chem. A.

[44] Buffet J.-C., Byles C.F.H., Felton R., Chen C., O’Hare D. (2016). Metallocene supported core@LDH catalysts for slurry phase ethylene polymerisation. Chem. Commun..

[45] Chen C., Yee L.K., Gong H., Zhang Y., Xu R. (2013). A facile synthesis of strong near infrared fluorescent layered double hydroxide nanovehicles with an anticancer drug for tumor optical imaging and therapy. Nanoscale.

[46] Mantovani K.M., Molgero Westrup K.C., da Silva Junior R.M., Jaerger S., Wypych F., Nakagaki S. (2018). Oxidation catalyst obtained by the immobilization of layered double hydroxide/Mn(iii) porphyrin on monodispersed silica spheres. Dalton Trans..

[47] Tan X., Liu S., Liu Y., Gu Y., Zeng G., Cai X., Yan Z., Yang C., Hu X., Chen B. (2016). One-pot synthesis of carbon supported calcined-Mg/Al layered double hydroxides for antibiotic removal by slow pyrolysis of biomass waste. Sci. Rep..

[48] Pan D., Zhang H., Fan T., Chen J., Duan X. (2011). Nearly monodispersed core-shell structural Fe3O4@DFUR-LDH submicro particles for magnetically controlled drug delivery and release. Chem. Commun..

[49] Mandal S., Mayadevi S. (2008). Cellulose supported layered double hydroxides for the adsorption of fluoride from aqueous solution. Chemosphere.

[50] Yu L., Zhou H., Sun J., Qin F., Yu F., Bao J., Yu Y., Chen S., Ren Z. (2017). Cu nanowires shelled with NiFe layered double hydroxide nanosheets as bifunctional electrocatalysts for overall water splitting. Energy Environ. Sci..

[51] Zhang H., Pan D., Zou K., He J., Duan X. (2009). A novel core-shell structured magnetic organic-inorganic nanohybrid involving drug-intercalated layered double hydroxides coated on a magnesium ferrite core for magnetically controlled drug release. J. Mater. Chem..

[52] Soleimani Dorcheh A., Abbasi M.H. (2008). Silica aerogel; synthesis, properties and characterization. J. Mater. Process. Technol..

[53] Maleki H., Durães L., Portugal A. (2014). An overview on silica aerogels synthesis and different mechanical reinforcing strategies. J. Non-Cryst. Solids.

[54] Xia K., Lang W.-Z., Li P.-P., Long L.-L., Yan X., Guo Y.-J. (2016). The influences of Mg/Al molar ratio on the properties of PtIn/Mg(Al)O-x catalysts for propane dehydrogenation reaction. Chem. Eng. J..

[55] Du X., Zhang D., Shi L., Gao R., Zhang J. (2013). Coke-and sintering-resistant monolithic catalysts derived from in situ supported hydrotalcite-like films on Al wires for dry reforming of methane. Nanoscale.

[56] Tomar R., Singh N., Rathee G., Kumar N., Tomar V., Chandra R. (2017). Synthesis and Characterization of Hybrid Mg(OH)(2) and CeCO_3_OH Composite with Improved Activity Towards Henry Reaction. Asian J. Org. Chem..

[57] Feng X., Wang L., Yao X., Dong H., Wang X., Wang Y. (2017). Trace water/amino-modified silica aerogel catalytic system in the one-pot sequential reaction of benzaldehyde dimethyl acetal and nitromethane. Catal. Commun..

[58] Tang Y., Gu X.F., Meng M., Xu J.F. (2013). Direct Henry reactions with modified calcium oxide as solid catalyst. Res. Chem. Intermed..

[59] Akutu K., Kabashima H., Seki T., Hattori H. (2003). Nitroaldol reaction over solid base catalysts. Appl. Catal. A-Gen..

[60] Choudary B.M., Kantam M.L., Reddy C.V., Rao K.K., Figueras F. (1999). Henry reactions catalysed by modified Mg-Al hydrotalcite: An efficient reusable solid base for selective synthesis of beta-nitroalkanols. Green Chem..

[61] Huang J.P., Li C.M., Tao L.L., Zhu H.L., Hu G. (2017). Synthesis, characterization and heterogeneous base catalysis of amino functionalized lanthanide metal-organic frameworks. J. Mol. Struct..

[62] Komura K., Kawamura T., Sugi Y. (2007). Layered silicate PLS-1: A new solid base catalyst for C-C bond forming reactions. Catal. Commun..

[63] Motokura K., Viswanadham N., Dhar G.M., Iwasawa Y. (2009). Creation of acid-base bifunctional catalysis for efficient C-C coupling reactions by amines immobilization on SiO_2_, silica-alumina, and nano-H-ZSM-5. Catal. Today.

[64] He Y.X., Jawad A., Li X., Atanga M., Rezaei F., Rownaghi A.A. (2016). Direct aldol and nitroaldol condensation in an aminosilane-grafted Si/Zr/Ti composite hollow fiber as a heterogeneous catalyst and continuous-flow reactor. J. Catal..

[65] Shaikh M., Sahu M., Gavel P.K., Turpu G.R., Khilari S., Pradhan D., Ranganath K.V.S. (2016). Mg-NHC complex on the surface of nanomagnesium oxide for catalytic application. Catal. Commun..

[66] Thangaraj B., Jayaraj C., Srinivasan R., Ayyamperumal S. (2015). Diaminosilane-functionalized on silicate-stabilised hydrotalcite (MA-HTSi-DA): As potential catalyst for nitro-aldol condensation. J. Mol. Catal. A-Chem..

