# Supplementary material for: Small-Sized Mg–Al LDH Nanosheets Supported on Silica Aerogel with Large Pore Channels: Textural Properties and Basic Catalytic Performance after Activation
Source: Nanomaterials (Basel). 2018 Feb 16;8(2):113. doi: 10.3390/nano8020113 (PMC5853744; doi:10.3390/nano8020113)
Supplement: Supplementary file 1 [file nanomaterials-08-00113-s001.pdf]

Supporting Information

Small-sized Mg-Al LDH nanosheets supported on silica aerogel with large pore channels: Textural properties and basic catalytic performance after activation

Lijun Wang *, Yusen Wang, Xiaoxia Wang, Xiaolan Feng, Xiao Ye, Jie Fu

School of Chemistry and Chemical Engineering, Shaoxing University, Shaoxing 312000, PR China

*Corresponding author. E-mail address: ljwang@usx.edu.cn

Figures and Tables

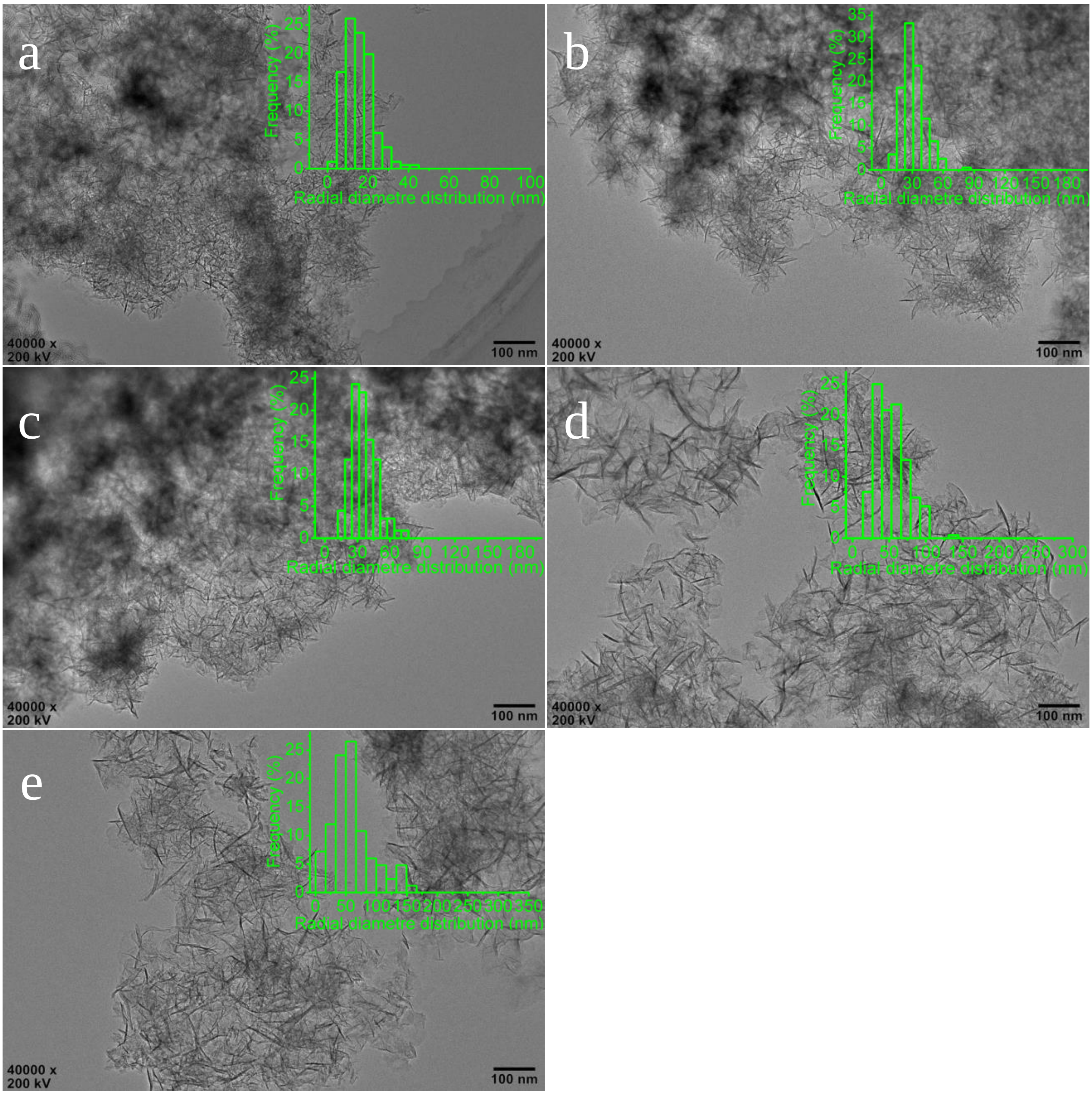

Figure S1 TEM images of SA/LDH-Mg_2Al-80 (a), SA/LDH-Mg_5Al_2-80 (b), SA/LDH-Mg_3Al-80 (c), SA/LDH-Mg_2Al-105 (d) and SA/LDH-Mg_2Al-150 (e) with a large length-to-height ratio of 1.5; the insets are the radial diameter distributions of the supported LDH nanosheets acquired from >150 nanoparticles in the corresponding images.

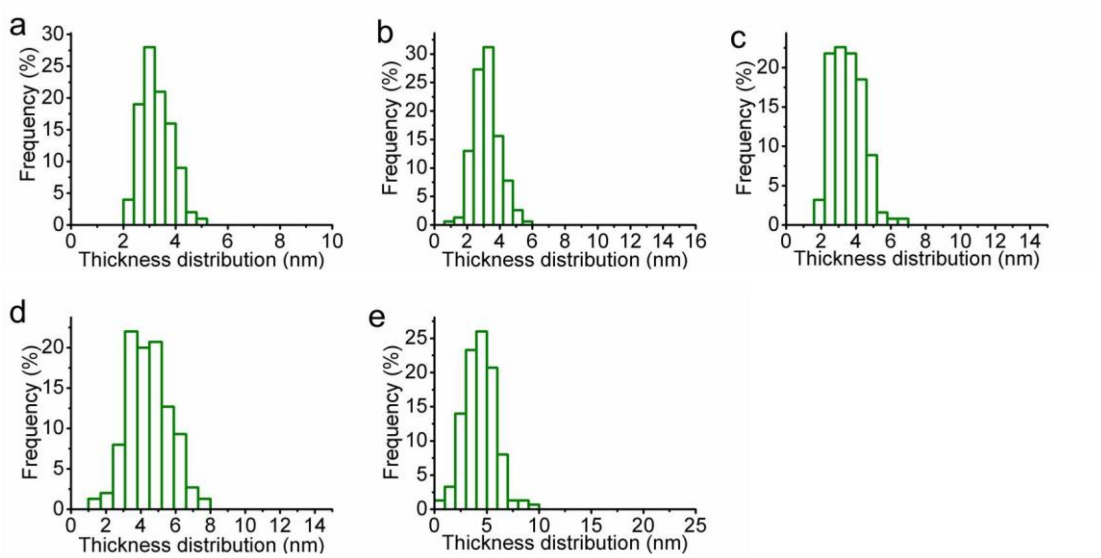

Figure S2 Thickness distributions of the supported LDH nanosheets: SA/LDH-Mg_2Al-80 (a), SA/LDH-Mg_5Al_2-80 (b), SA/LDH-Mg_3Al-80 (c), SA/LDH-Mg_2Al-105 (d) and SA/LDH-Mg_2Al-150 (e) acquired from >150 nanoparticles in Figure S1.

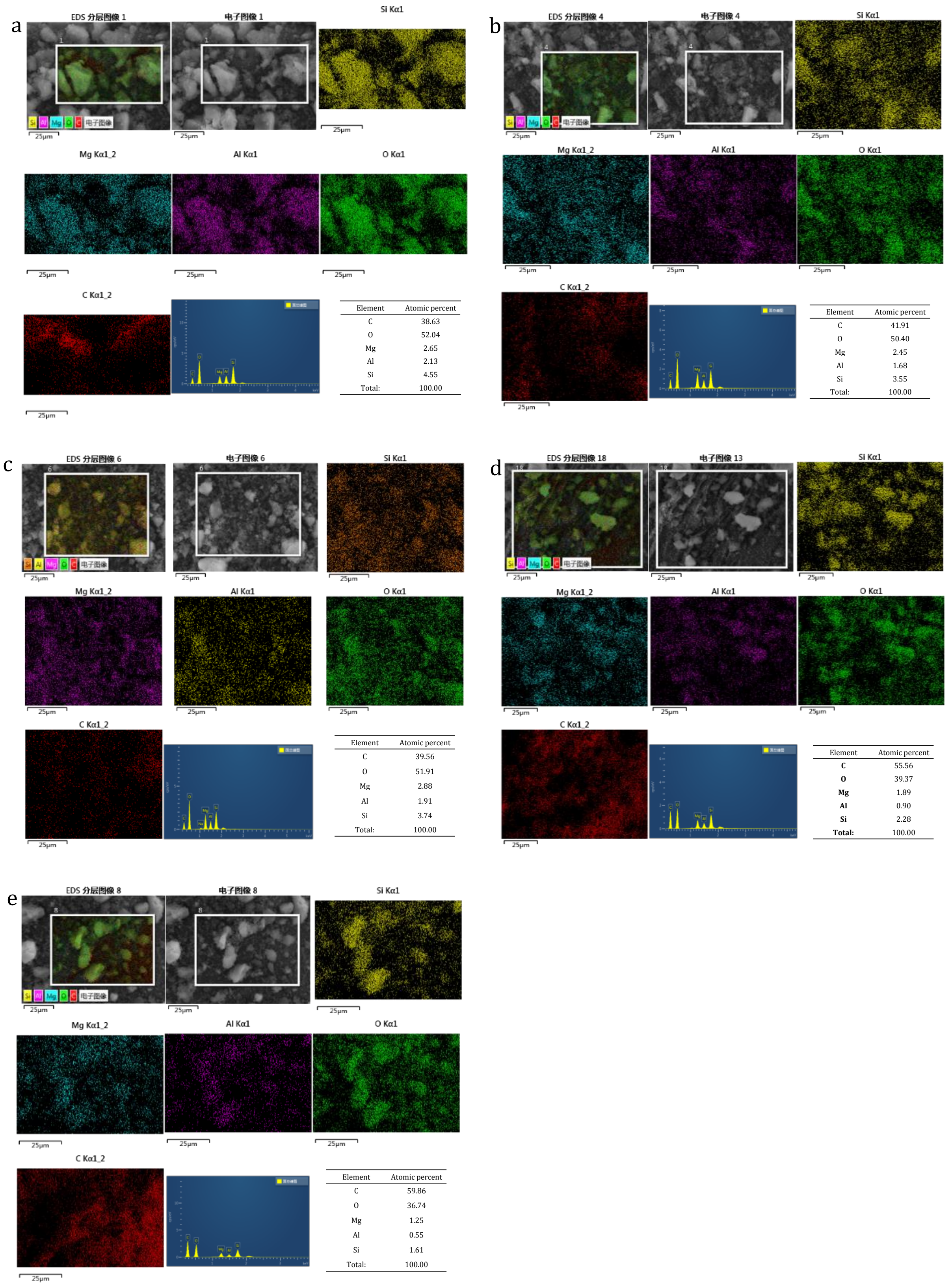

Figure S3 EDS mappings and corresponding elemental analysis of SA/LDH-Mg_2Al-80 (a), SA/LDH-Mg_2Al-105 (b), SA/LDH-Mg_2Al-150 (c), SA/LDH-Mg_5Al_2-80 (d) and SA/LDH-Mg_3Al-80 (e).

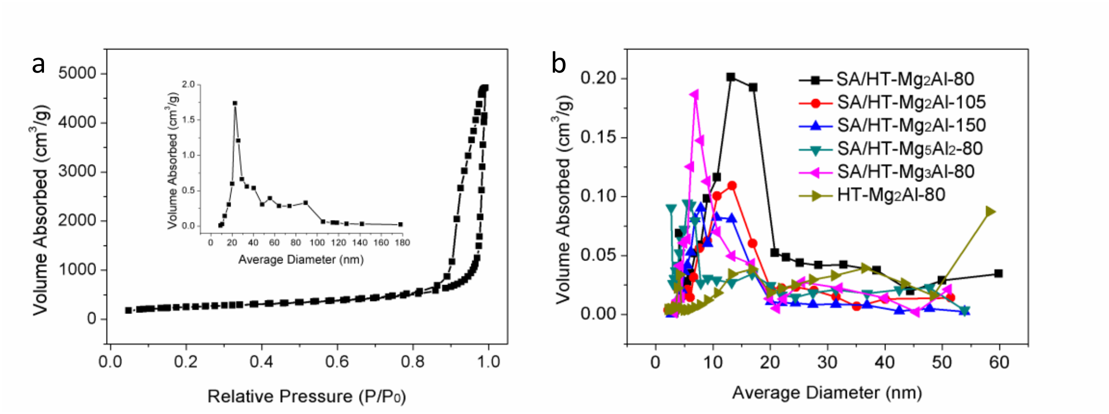

Figure S4 (a) N_2 adsorption−desorption isotherms and the corresponding BJH pore size distribution (inset) of SA; (b) BJH pore size distributions of the SA/LDH series and the unsupported LDH (LDH-Mg_2Al-80).

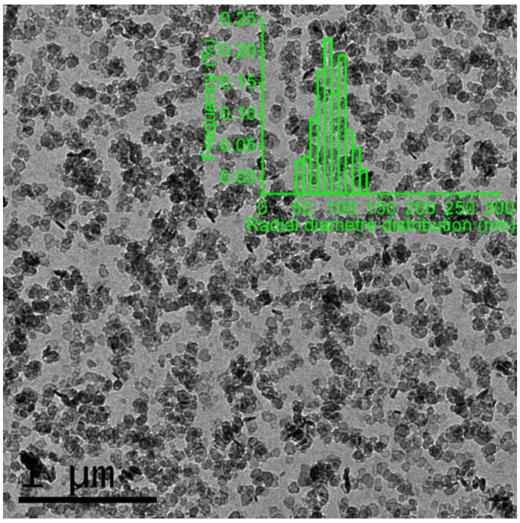

Figure S5 TEM image of unsupported LDH nanosheets (LDH-Mg_2Al-80) using as a contrast sample. The inset is the corresponding radial diameter distribution.

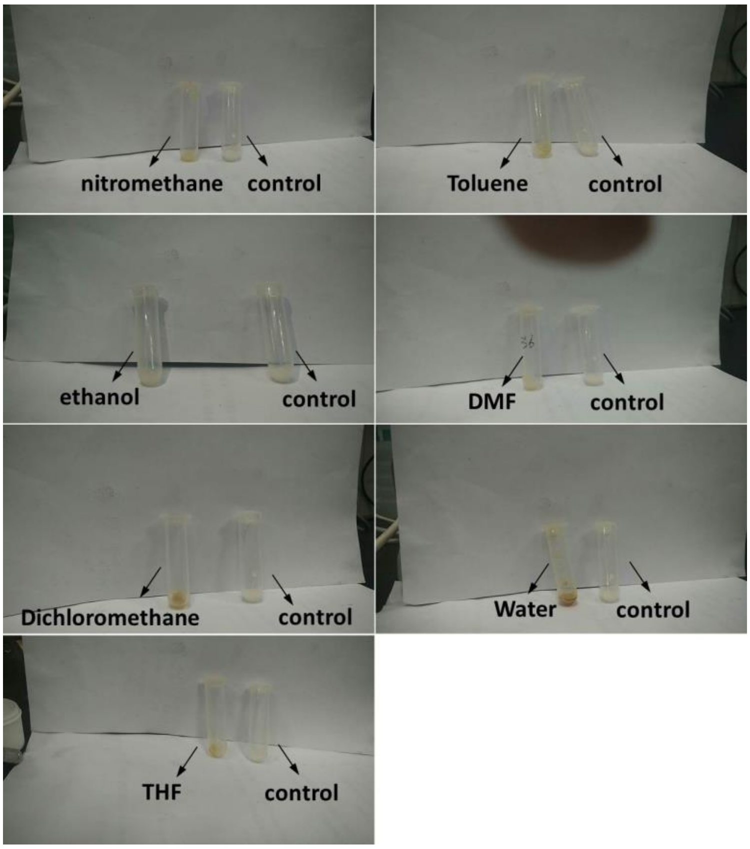

Figure S6 The used catalyst SA/LDH-Mg_2Al-80 collected by centrifugation after the reaction of benzaldehyde with nitromethane in different solvents such as nitromethane, ethanol, dichloromethane, toluene, DMF, Water and THF.

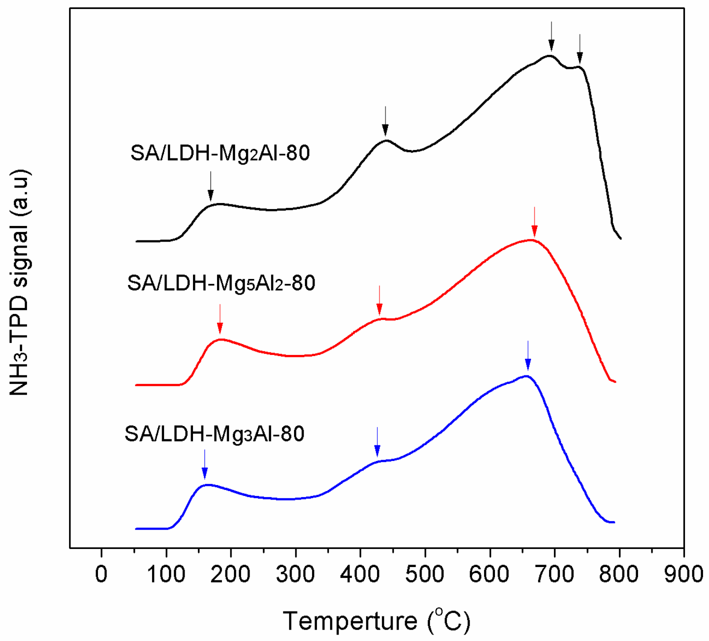

Figure S7 NH_3-TPD profiles of SA/LDH series synthesized at a temperature of 80°C.

Table S1 The semi-quantitative results of NH_3-TPD measurements. [a]

Samples	NH_3-TPD peak position (°C)			Total peak area (a.u.) [a]
	I	II	III	
SA/LDH-Mg_2Al-80	168.0	440.0	695.5/736.4	99705.9
SA/LDH-Mg_5Al_2-80	183.6	431.8	667.8	80575.0
SA/LDH-Mg_3Al-80	161.0	429.1	660.6	83856.0

[a] Total peak area is linearly proportional to the amount of NH_3 adsorbed.
